# The Association Between Exposure to Low Magnesium Blood Levels After Renal Transplantation and Cardiovascular Morbidity and Mortality

**DOI:** 10.3389/fmed.2021.690273

**Published:** 2021-07-12

**Authors:** Itay Lahav, Tali Steinmetz, Maya Molcho, Neta Lev, Timna Agur, Eviatar Nesher, Benaya Rozen-Zvi, Ruth Rahamimov

**Affiliations:** ^1^Sackler School of Medicine, Tel Aviv University, Tel Aviv, Israel; ^2^Department of Nephrology, Rabin Medical Center, Petah Tikva, Israel; ^3^Department of Organ Transplantation, Rabin Medical Center-Beilinson Hospital, Petah Tikva, Israel

**Keywords:** magnesium, renal transplantation, cardiovascular outcomes, mortality, renal function

## Abstract

**Background:** Serum magnesium levels are associated with cardiovascular disease and all-cause mortality in the general population and chronic kidney disease patients, but the association between serum magnesium levels and cardiovascular risk after kidney transplantation is not established. We sought to evaluate whether exposure to low serum magnesium levels after renal transplantation is related to cardiovascular morbidity and mortality.

**Methods:** We conducted a single center retrospective study that included all transplanted patients who had a functioning graft for at least 6 months after transplantation between January 2001 and December 2013. We calculated exposure to magnesium using time weighted average for serum magnesium levels, using all values available during the follow-up. Several statistical methods were used, including liner regression analysis, χ^2^ test, and multivariate Cox proportional hazard model.

**Results:** Four hundred ninety-eight patients were included. Median follow-up was 5.26 years. High time weighted average of serum magnesium was associated with a hazard ratio of 1.94 for all-cause mortality and major cardiovascular outcome compared to low levels (95% CI 1.18–3.19, *p* = 0.009). The high quartile of time weighted average of serum magnesium was associated with death censored major cardiovascular outcome (hazard ratio 2.13, 95% CI 1.17–3.86, *p* = 0.013) in multivariate analysis.

**Conclusions:** Exposure to low serum magnesium levels in renal transplant recipients was associated with a lower risk for all-cause mortality and major cardiovascular outcome. These findings contrast the higher risk found in the general population.

## Introduction

Magnesium (Mg) is the second most prevalent intracellular cation (1) and has a vital role in cellular and biological processes. Serum magnesium (sMg) levels depend on the balance between renal excretion and intestinal absorption; thus, hypomagnesemia results from gastrointestinal losses and renal losses due to medications such as loop or thiazide-type diuretics, calcineurin inhibitors and cisplatin, hypercalcemia, volume expansion, recovering acute tubular necrosis, post-obstructive diuresis and inherited tubulopathies (e.g., Bartter and Gitelman syndromes) (2).

Data from epidemiological studies suggests that low sMg levels are associated with increased cardiovascular disease (CVD) and all-cause mortality in the general population (3, 4).

In chronic kidney disease (CKD) patients, there is an increasing body of evidence for an inverse association between sMg and insulin resistance, new onset diabetes (5), hypertension (6), atherosclerosis (7), inflammation (8), CVD (3, 9), vascular calcification (10), dyslipidemia (11), and renal function decline and mortality risk (12, 13).

Magnesium deficiency is common after kidney transplantation mainly due to the effect of calcineurin inhibitors (CNI) on tubular magnesium handling. CNI induce hypomagnesemia through downregulation of the renal expression of epidermal growth factor (EGF) and transient receptor potential channel melastatin 6 (TRMP6) in the distal collecting tubule, leading to Mg wasting (14). Hypomagnesemia is associated with new onset diabetes after transplantation (15, 16) and also with vascular stiffness and endothelial dysfunction (17), measured by carotid-femoral pulse wave velocity. The association between sMg levels and CVD in kidney transplant recipients has not been evaluated yet.

The aim of this study was to evaluate whether there is association between exposure to low magnesium blood levels after renal transplantation and cardiovascular morbidity and mortality.

## Materials and Methods

### Study Design

This is a single-center retrospective cohort study. The study protocol was approved by the Rabin Medical Center (RMC) institutional review board (IRB).

### Study Population

**Figure 1:**
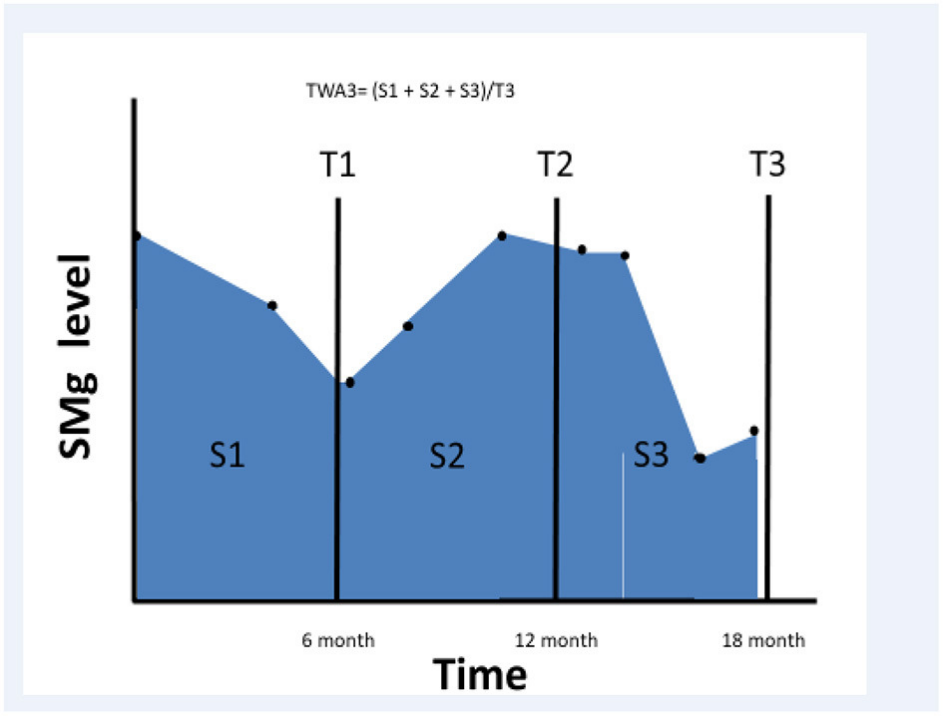
Schematic description of sMg TWA calculation.

We searched the RMC kidney transplant registry between January 2001 and December 2013 and selected all transplanted patients who had a functioning graft for at least 6 months after transplantation. We collected sMg values and calculated time weighted average (TWA) for sMg levels using all values available since the renal transplantation and during the follow-up period. TWA was calculated by multiplying each sMg value by the time of exposure, summing all the values, and dividing them by the time interval between the first and last values. We evaluated cumulative TWA of sMg from time of transplantation for each time point, by calculating TWA between the time of transplantation to the end of each 6-month interval. As a result, the exposure variable for this study was the mean sMg (weighted for time of exposure) during all the follow-up time until the time of the event. Schematic description of TWA for sMg at each time point is depicted in Figure 1.

We excluded patients with less than six magnesium blood level values available, patients who were lost to follow-up, multiple organ transplant patients, and patients who experienced cardiovascular events during the first 6 months after the transplantation.

### Endpoints

The primary endpoint was the composite outcome of all-cause mortality and major cardiovascular outcome (MACE) defined as non-fatal myocardial infarction (MI), non-fatal cerebrovascular accident (CVA), coronary revascularization, peripheral revascularization, and hospitalization due to acute coronary syndrome, congestive heart failure (CHF) exacerbation, or peripheral vascular disease (PVD).

Secondary endpoints included the separate components of the primary outcome, graft survival, and overall survival. The outcome of MACE was determined by two physicians (I.V. and M.M.). The physicians opened every medical file and did not rely on the written diagnosis. Whenever there was a doubt about the diagnosis, the physicians consult with a senior nephrologist (R.R. or B.R.Z.) in order to ascertain the MACE event. In any disagreement, another senior nephrologist (R.R. or B.R.Z.) was consulted.

### Study Design

**Figure 2:**
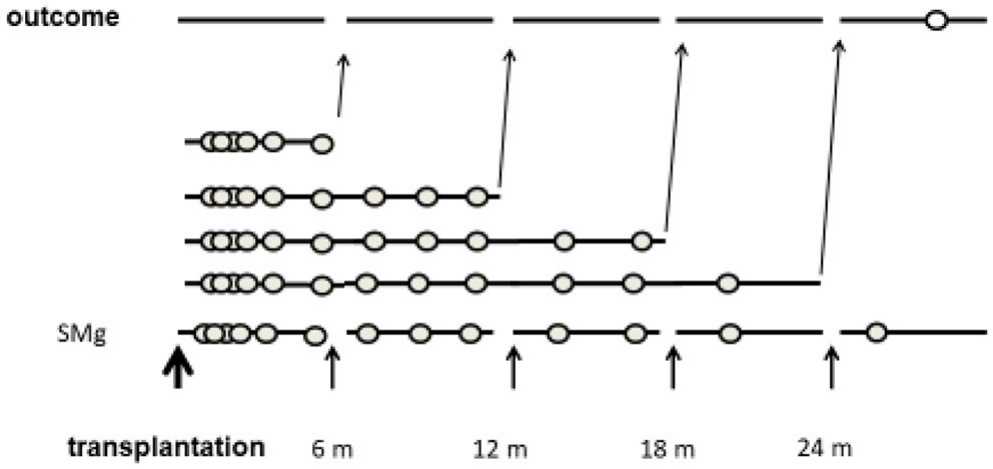
Schematic description of study design.

The time period from transplantation was divided into 6-month intervals. The primary exposure was TWA of sMg from the transplantation to the end of each interval. Each patient was assigned into a group according to the TWA of sMg at the beginning of each interval. As the TWA of sMg changed over time, each patient could be assigned to a different group at each time interval. Each outcome episode was attributed to the TWA of SMg calculated from the transplantation to the end of the previous time interval, and analysis was done by time-varying manner. A schematic description of the study design is depicted in Figure 2.

### Statistical Methods

Baseline patient characteristics are presented as means and standard deviations or median and interquartile range, as appropriate.

Data was collected from the patients' electronic health records. Recipient data included age, gender, baseline kidney disease, body mass index (BMI), history of hypertension, diabetes mellitus, ischemic heart disease, MI, coronary revascularization, CHF, valvular disease, PVD, CVA, past renal replacement therapy, and transplantations. Transplantation data included donor type (living or deceased), age and gender, panel reactive antibodies (PRA) status, human leukocyte antigen (HLA) mismatch, induction therapy, maintenance immunosuppression therapy, delayed graft function, hospitalization length, and episodes of acute rejections.

Laboratory data was collected at 6 months post-transplantation and included creatinine, urea, low-density lipoprotein (LDL) cholesterol, glucose, cholesterol, albumin, hemoglobin, white blood cells (WBC), platelets, and hemoglobin A1C (HBA1C). At 6 months, we also evaluated for development of hypertension and diabetes and recorded medications prescribed including immunosuppression medication (prograf, cyclosporine, mTOR inhibitors), magnesium supplements, blood pressure medications (ACE inhibitors, Ca channel blockers, Beta blockers), anti-aggregates, statins, and proton pump inhibitors. The protocol for magnesium supplementation at our clinic was magnesium citrate 100 mg thrice daily, and it was given at the discretion of the treating physician.

We used multiple imputations using liner regression analysis, with five repeats for missing data analysis. Mean and standard deviation described normally distributed variables, whereas median and range described non-normally distributed variables. For differences between groups, we used analysis of variance (ANOVA) for continuous variables and χ^2^ test for discreet variables.

For modeling time to first event, we used time varying univariate and multivariate Cox proportional hazard model, with quartiles of TWA sMg as time dependent variable. We compared quartiles of sMg according to the TWA of sMg from the time of transplantation to each time point. The proportionality assumption was validated by evaluating each variable for interaction with time and assessing for the null hypothesis. For multivariate analysis, we used forward stepwise analysis with possible confounders using inclusion *p*-value of 0.05. We also forced into the model variables that were significantly associated with TWA of magnesium at 6 months post-transplantation. The variables in the multivariate analysis included recipient age and gender, BMI, history of heart disease, diabetes mellitus, smoking status, donor age, Mg supplementation, maintenance immunosuppression therapy, duration of dialysis, serum albumin, and eGFR. The full models of the multivariate analysis are presented in Supplementary Tables 1, 3, 6.

In order to further evaluate the risk of our primary and secondary outcomes, we used repeated measures generalized estimating equations (GEE). We used the cumulative TWA of magnesium level at the beginning of each time interval as repeated measures variable and the odds for an event during each interval as response variable using binary logistic model. The multivariable adjusted model included the same variables used for the time varying Cox analysis.

In order to increase the internal validity of our results, we performed several sensitivity analyses. We analyzed the association between sMg level and the primary outcome in all the patients in the cohort including patients with <6 sMg values and patients that had CV event during the first 6 months post-transplantation. We also analyzed the association between the highest sMg value and the primary outcome. Lastly, we evaluated the association between TWA of sMg at 1 year and the secondary outcome of death censored CV events using competing risk analysis with non-CV death as the competing risk.

## Results

**Figure 3:**
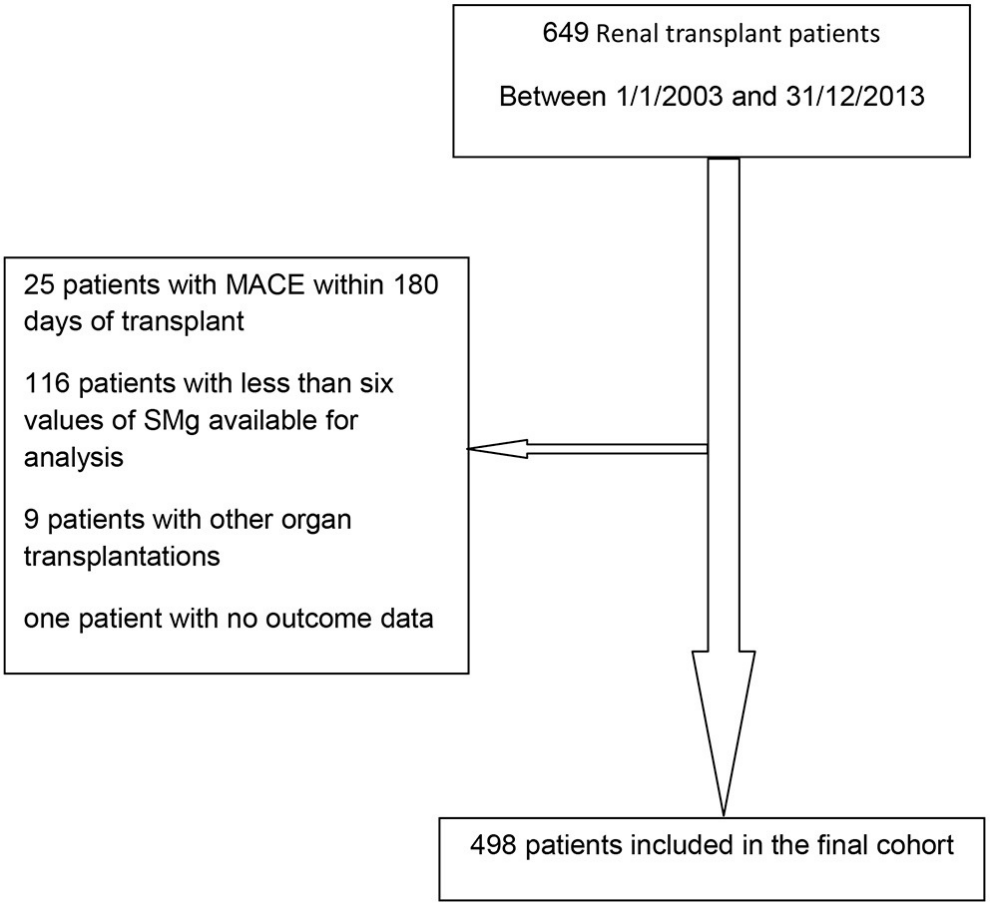
Flow diagram of the recruitment and exclusion of patients in the study.

Between January 1, 2003 and December 31, 2013, 649 patients were transplanted; 498 were included in the final cohort. Reasons for exclusion were as follows: MACE within 180 days of transplant (18), less than six values of sMg available for analysis (116), other organ transplantations (9), and no outcome data (1). Flow diagram of the recruitment and exclusion of patients in the study is depicted in Figure 3.

The patients' characteristics according to TWA of magnesium concentration during the first 6 months post-transplantation are presented in Table 1. Low TWA of serum magnesium was associated with younger donor age, higher estimated glomerular filtrate rate (eGFR), treatment with magnesium supplementation, and non-smoker status.

**Table 1:** Baseline characteristics of the entire study population according to TWA quartiles of sMg during the first 6 months post-transplantation.

	**Quartile 1 (*n* = 123)**	**Quartile 2 (*n* = 121)**	**Quartile 3 (*n* = 122)**	**Quartile 4 (*n* = 127)**	***P*-value**
**Baseline variables**					
Age (years)	47.1 ± 15.3	49.1 ± 14.4	47.9 ±14.5	47.7 ± 15	0.765
Gender (men)	50 (40.7%)	38 (31.4%)	40 (32.8%)	45 (35.4%)	
Dialysis duration (months)	35.6 ± 33.4	34.7 ± 40.2	30.3 ± 35.8	31.9 ± 32.9	0.633
Living donor	68 (55.3%)	64 (52.9%)	79 (64.8%)	76 (59.8%)	0.248
Donor age	40.7 ± 15.3	44.1 ± 14.3	45.5 ± 12.5	49.6 ± 13	<0.001
Systolic blood pressure	131.2 ± 19.4	129.3 ± 17.9	132.4 ± 18.5	130.1 ± 17.9	0.663
Creatinine at 6 months	1.26 ± 0.43	1.45 ± 0.67	1.45 ± 0.48	1.45 ± 0.48	0.005
eGFR at 6 months	66.74 ± 21.49	58.25 ± 18.38	57.47 ± 18.99	56.17 ± 19.68	<0.001
BMI	26.34 ± 5.9	25.89 ± 5.4	26.76 ± 5.3	25.37 ± 4.9	0.303
Triglycerides	178.3 ± 86.2	177.4 ± 130.1	171.7 ± 111.5	173.7 ± 86.8	0.957
LDL	92.5 ± 23.3	90.4 ± 25.9	91.8 ± 23.7	86.9 ± 27.9	0.331
Calcium	9.9 ± 0.76	9.8 ± 0.6	9.8 ± 0.6	9.8 ± 0.6	0.688
HDL	50.1 ± 14.3	49.8 ± 14.5	51.2 ± 13.7	51.2 ± 14.9	0.822
Phosphorus	3.2 ± 0.87	3.2 ± 0.76	3.2 ± 0.72	3.4 ± 0.74	0.099
Albumin	4.2 ± 0.36	4.3 ± 0.33	4.3 ± 0.35	4.3 ± 0.4	0.749
Cholesterol	177.7 ± 30.2	175 ± 33.2	176.9 ± 34.8	171.9 ± 35.5	0.535
Glucose	119.8 ± 48.1	112.8 ± 53	116.2 ± 45.4	117.9 ± 60.9	0.787
Mg at 6 months	1.55 ± 0.08	1.68 ± 0.09	1.77 ± 0.03	1.95 ± 0.14	<0.001
Mg at 1 year	1.6 ± 0.08	1.72 ± 0.09	1.82 ± 0.07	1.97 ± 0.15	<0.001
Gender	73 (59.3%)	83 (68.6%)	82 (67.2%)	82 (64.6%)	0.443
Diabetes before transplantation	35 (28.5%)	33 (27.3%)	34 (27.9%)	42 (33.1%)	0.735
Diabetes at 6 months	47 (38.2%)	41 (33.9%)	44 (36.1%)	52 (40.9%)	0.694
IHD before transplantation	17 (13.8%)	27 (22.3%)	31 (25.4%)	29 (22.8%)	0.132
Transplantation number (*n* = 2)	14 (11.4%)	10 (8.3%)	7 (5.7%)	18 (14.2%)	0.132
Delate graft function	21 (17.1%)	24 (19.8%)	26 (21.3%)	35 (27.6%)	0.222
Hypertension	80 (65%)	74 (61.2%)	77 (63.1%)	85 (66.9%)	0.593
Prograf	118 (95.9%)	113 (93.4%)	107 (87.7%)	116 (91.3%)	0.129
Cyclosporine	3 (2.4%)	7 (5.8%)	7 (5.7%)	6 (4.7%)	
mTOR inhibitors	2 (1.6%)	1 (0.8%)	8 (6.6%)	5 (3.9%)	
Anti-aggregates	96 (78%)	96 (80%)	104 (85.2%)	105 (82.7%)	0.493
PPI	93 (75.6%)	89 (73.6%)	88 (72.1%)	91 (71.7%)	0.714
ARB/ACE inhibitors	21 (17.1%)	21 (17.6%)	28 (23%)	33 (26%)	0.247
Mg supplement	32 (26.2%)	19 (15.8%)	9 (7.4%)	6 (4.7%)	<0.001
Calcium supplement	21 (17.1%)	18 (15%)	13 (10.7%)	18 (14.2%)	0.542
Phosphorus supplement	8 (6.5%)	4 (3.3%)	6 (4.9%)	5 (3.9%)	0.664
Statins	62 (50.4%)	54 (45%)	74 (60.7%)	62 (48.8%)	0.088
Beta-blockers	60 (48.8%)	49 (40.8%)	70 (57.4%)	57 (44.9%)	0.063
Calcium channel blockers	45 (36.6%)	40 (33.6%)	56 (45.9%)	56 (44.4%)	0.144
Acute coronary syndrome	11 (8.9%)	16 (13.2%)	7 (5.7%)	6 (4.7%)	0.065
Any smoking	30 (24.4%)	48 (39.7%)	47 (38.5%)	51 (40.2%)	0.027
Current smoking	11 (8.9%)	26 (21.5%)	17 (13.9%)	18 (14.2%)	0.05

### Time Varying TWA of Blood Magnesium and the Primary Outcome

During a median follow-up time of 5.26 years [interquartile range (IQR) 4.21–7.37], 145 outcome events occurred including 98 events of MACE and 47 events of death.

We divided the patients into quartiles according to TWA of sMg at each time point. Table 2 presents the association between time varying TWA of Mg levels and the primary composite outcome of MACE and overall death.

**Table 2:** Association between TWA quartiles of Mg level and the primary composite outcome of MACE and overall death by univariate and multivariate analysis.

	**Univariate analysis**			**Multivariate analysis**		
	**Hazard ratio**	**95% confidence interval**	***P*-value**	**Hazard ratio**	**95% confidence interval**	***P*-value**
Quartile 1	Ref	Ref	Ref	Ref	Ref	Ref
Quartile 2	1.51	0.89–2.56	0.124	1.86	1.08–3.18	0.024
Quartile 3	1.57	0.93–2.54	0.09	1.73	0.99–3.01	0.054
Quartile 4	1.94	1.18–3.19	0.009	1.86	1.08–3.21	0.027

When compared to the lowest quartile, the highest quartile was associated with increased risk of the primary outcome [Hazard Ratio (HR) 1.94, 95% Confidence Interval (CI) 1.18–3.19, *p* = 0.009]. The results were not significantly changed by multivariate adjustment (HR 1.86, 95% CI 1.08–3.21, *p* = 0.027). We also analyzed the data by repeated measures GEE with comparable results [Odd Ratio (OR) 1.69, 95% CI 1.03–2.77, *p* = 0.037] and (OR 1.8, 95% CI 1.04–3.1, *p* = 0.034) for univariate and adjusted analysis, respectively (see Supplementary Table 2). Sensitivity analysis using the highest sMgl level gave similar results (HR 2.27, 95% CI 1.331–3.872, *p* = 0.003) and (HR 1.882, 95% CI 1.088–3.255, *p* = 0.024) for the highest quartile vs. the lowest quartile by univariate and multivariate analysis, respectively (see Supplementary Table 4).

Another sensitivity analysis included patients with <6 sMg levels (but at least one value) and patients that had CV events during the first 6 months post-transplantation. This analysis showed comparable results though, with numerically smaller effect size, when compared to the lowest quartile; the highest quartile was associated with increased risk of the primary outcome (HR 1.528, 95% CI 1.001–2.334, *p* = 0.05). The results were not significantly changed by multivariate adjustment (see Supplementary Table 5).

Comparable results were obtained when the secondary outcome of death censored MACE (with the exception of CV mortality) was evaluated. The high quartile of TWA of magnesium was associated with this outcome (HR 1.84, 95% CI 1.07–3.18, *p* = 0.028) and (HR 2.13, 95% CI 1.17–3.86, *p* = 0.013) for univariate and multivariate analysis, respectively. The results of association between sMg quartiles and death censored MACE and the full multivariate model are presented in Table 3 and Supplementary Table 6, respectively.

**Table 3:** Association between sMg quartiles and death censored MACE by univariate and multivariate analysis.

	**Univariate analysis**			**Multivariate analysis**		
	**Hazard ratio**	**95% confidence interval**	***P*-value**	**Hazard ratio**	**95% confidence interval**	***P*-value**
Quartile 1	Ref	Ref	Ref	Ref	Ref	Ref
Quartile 2	1.44	0.81–2.54	0.213	1.76	0.98–3.17	0.059
Quartile 3	1.09	0.59–2.2	0.781	1.38	0.72–2.73	0.332
Quartile 4	1.84	1.07–3.18	0.028	2.13	1.18–3.86	0.013

When the association with death censored MACE was analyzed by competing risk analysis (with the competing risk of non-CV death), the highest quartile of TWA of Mg at 1 year was non-significantly associated with CVD by univariate analysis (HR 1.47, 95% CI 0.93–2.33, *p* = 0.17). However, by multivariate analysis, the association was significant (HR 1.88, 95% CI 1.14–3.11, *p* = 0.038) (see Supplementary Table 7).

In a similar manner, analysis by repeated measures GEE generated equivalent results (OR 1.69, 95% CI 0.97–2.94, *p* = 0.062) and (OR 2.15, 95% CI 1.15–4.02, *p* = 0.017) for univariate and adjusted analysis, respectively. As can be seen, the trend for increased odds for CV events was non-significant by univariate analysis but became significant after multivariate adjustment (see Supplementary Table 8).

Table 4 presents the association between time varying TWA of serum magnesium and the separate components of MACE.

**Table 4:** Association between quartiles of TWA of sMg and the separate components of MACE.

		**Hazard ratio**	**95% confidence interval**		***P*-value**
ACS	q1	ref	ref	ref	ref
	q2	0.782	0.324	1.889	0.585
	q3	0.528	0.195	1.429	0.209
	q4	0.858	0.364	2.021	0.726
CVA	q1	ref	ref	ref	ref
	q2	1.264	0.283	5.648	0.759
	q3	1.959	0.49	7.835	0.342
	q4	2.815	0.762	10.403	0.121
PVD	q1	ref	ref	ref	ref
	q2	1.56	0.731	3.331	0.25
	q3	0.898	0.381	2.115	0.805
	q4	1.583	0.753	3.327	0.225
CHF	q1	ref	ref	ref	ref
	q2	0.877	0.338	2.276	0.788
	q3	1.534	0.655	3.595	0.324
	q4	1.406	0.601	3.293	0.432
CV DEATH	q1	ref	ref	ref	ref
	q2	0.794	0.213	2.959	0.731
	q3	1.025	0.297	3.543	0.969
	q4	1.306	0.414	4.117	0.648

### Time Varying TWA of Blood Magnesium and All-Cause Mortality

During the follow-up, 77 patients died: 22 (28.6%) due to CVD, 30 (39%) due to infections, 7 (9.1%) due to malignancy, and 18 (23.4%) due to other or unknown cause. Table 5 presents the association between quartiles of TWA of sMg levels during the follow-up and the secondary outcome of all-cause mortality. By univariate analysis, both the third and fourth quartiles were associated with increased mortality compared to the lowest quartile (HR 2.2, 95% CI 1.08–4.49, *p* = 0.031) and (HR 2.16, 95% CI 1.06–4.38, *p* = 0.034), respectively. By multivariate analysis, only the third quartile was significantly associated with mortality, while there was only a non-significant trend for the fourth quartile (Table 5). The full model of the multivariate analysis is presented in Supplementary Table 6.

**Table 5:** Association between TWA quartiles of Mg and all-cause mortality by univariate and multivariate analysis.

	**Univariate analysis**			**Multivariate analysis**		
	**Hazard ratio**	**95% confidence interval**	***P*-value**	**Hazard ratio**	**95% confidence interval**	***P*-value**
Quartile 1	Ref	Ref	Ref	Ref	Ref	Ref
Quartile 2	1.45	0.67–3.13	0.343	1.59	0.73–3.45	0.241
Quartile 3	2.2	1.08–4.49	0.031	2.37	1.14–3.91	0.021
Quartile 4	2.16	1.06–4.38	0.034	1.94	0.93–4.06	0.079

By repeated measures GEE, univariate analysis the fourth quartile was associated with increased mortality compared to the lowest quartile with non-significant trend for the third quartile (OR 2.18, 95% CI 1.08–4.41, *p* = 0.029 and OR 1.9, 95% CI 0.93–3.89, *p* = 0.081, respectively). By multivariate analysis, the association was no longer significant for both quartiles compared to the lowest quartile (OR 1.74, 95% CI 0.85–3.55, *p* = 0.127 and OR 1.94, 95% CI 0.94–4.02, *p* = 0.074 for the third and fourth quartiles, respectively) (see Supplementary Table 9).

### Association of Magnesium Supplementation at 6 Months and CV Outcomes

Of the 498 patients included in the study, 495 (99.4%) had information available regarding prescribed medications 6 months post-transplantation. Sixty-six of the 495 patients (13.3%) were treated with magnesium supplementation. Table 6 describes the characteristics of patients treated with magnesium supplementation vs. the untreated patients.

**Table 6:** Baseline characteristics of patients treated with magnesium supplementation vs. untreated patients.

	**No Mg supplement**	**Mg supplement**	***P*-value**
Number of patients	429	66	
Age	47.83 ± 14.79	49.00 ± 14.52	0.551
Gender (men)	278 (64.8%)	41 (62.1%)	0.672
Dialysis duration (months)	32.58 ± 36.49	37.09 ± 29.07	0.339
Donor age (years)	45.37 ± 13.93	43.52 ± 15.27	0.3
Systolic BP (mm Hg)	130.43 ± 18.95	131.23 ± 13.61	0.76
BMI (kg/m^2^)	26.08 ± 5.45	25.81 ± 6.42	0.747
LDL cholesterol	90.19 ± 25.21	91.02 ± 21.61	0.807
HDL cholesterol	50.66 ± 14.67	49.66 ± 11.86	0.601
Albumin	4.26 ± 0.37	4.32 ± 0.34	0.215
Corrected calcium	9.62 ± 0.56	9.83 ± 0.69	0.007
Glucose	116.49 ± 51.75	118.15 ± 54.59	0.817
eGFR (ml/min/1.73 m^2^)	58.53 ± 18.70	66.34 ± 13.42	0.003
Smoker status	61 (14.2%)	12 (18.2%)	0.398
History of IHD	93 (21.7%)	12 (18.2%)	0.518
Diabetes	159 (37.1%)	26 (39.4%)	0.716
Living donor	256 (59.7%)	32 (48.5%)	0.082
First transplantation	380 (88.6%)	64 (97.0%)	0.061
DGF	90 (17.1%)	16 (24.2%)	0.547
Hypertension	270 (63.2%)	49 (74.2%)	0.082
Prograf	392 (91.4%)	63 (95.5%)	0.395
Cyclosporine	23 (5.4%)	1 (1.5%)	
mTOR inhibitors	14 (3.3%)	2 (3.0%)	
Anti-aggregates	356 (83.0%)	48 (72.7%)	0.045
PPI	307 (71.6%)	56 (84.8%)	0.023
Ace inhibitors	97 (22.7%)	6 (9.1%)	0.018
Statins	217 (50.6%)	34 (51.5%)	0.888
Beta blockers	199 (46.4%)	39 (59.1%)	0.063
Ca channel blockers	173 (40.5%)	25 (37.9%)	0.684

By univariate Cox analysis, magnesium supplementation was not significantly associated with the primary outcome (HR 1.46, 95% CI 0.9–2.37, *p* = 0.103). However, by the multivariate model including TWA for sMg at 6 months, age, gender, BMI, ischemic heart disease (IHD) before transplantation, serum calcium, albumin and LDL, treatment with PPI, beta blockers, and inhibitors of the renin angiotensin, the association became significant (HR 2.07, 95% CI 1.23–3.46, *p* = 0.006).

## Discussion

Previous research has shown an association between sMg levels and renal and cardiometabolic outcomes in the general population and patients with CKD. Due to a lack of studies examining this association in patients with kidney transplantation, we sought to examine this association. Our results indicate that renal transplant patients within the lowest quartile of exposure to sMg levels had a 1.5 lower risk for the composite outcome of all-cause mortality and MACE compared to patients with higher levels of exposure. In addition, magnesium supplements did not improve the prognosis of renal transplant patients and were also associated with negative outcomes by multivariate analysis.

Mg is essential for vital cellular functions and is required as a co-factor for many enzymatic reactions. Mg regulates ion channels that participate in neuromuscular excitability and cell permeability and immune response (1). In addition, Mg has anti-atherosclerosis, anti-inflammatory, and antioxidant properties. Recent studies demonstrated an association between dysmagnesemia and poor outcomes in the general population (3, 4), CKD patients (8, 13), dialysis patients (12), and heart failure patients (19). In a population-based study that included 4,203 individuals, low Mg levels were associated with higher all-cause mortality and cardiovascular mortality (20). Van laecke et al. conducted a study with 1,650 CKD patients and a follow-up time of 5.1 years. In the study, hypomagnesemia predicted mortality and renal function decline in CKD patients. Patients with low (<1.8 mg/dl) vs. high (>2.2 mg/dl) serum Mg had a 61% increased mortality risk (adjusted HR 1.613, 95% CI 1.113–2.338, *p* < 0.0001) (13).

A recent prospective multicenter study (CRIC study) that was conducted by Lavinia Negrea et al. included 3,867 patients with CKD. The study evaluated the association between sMg level and cardiovascular events and all-cause mortality. This was the largest study made in CKD patients with a long follow-up of 14.6 years. The authors conclude that sMg level <1.9 and >2.1 mg/dl was associated with increased risk for all-cause mortality. Low sMg level was associated with incident atrial fibrillation but not with composite CVD events. This study is added to the growing body of evidence that propose an association between high sMg level and cardiovascular outcomes (21).

Sakaguchi et al. in a study that included 142,555 HD patients showed a J-shaped association between serum magnesium and all-cause and cardiovascular mortality from the lowest to highest sextile, with significantly higher mortality in sextiles 1–3 and 6 (22). In contrast, Angkananard et al. (19) conducted a systematic review and meta-analysis that included seven prospective studies with a total of 5,172 chronic heart failure (CHF) patients. The meta-analysis suggested that in CHF patients, hypermagnesemia with Mg >/= 1.05 mmol/liter (2.55 mg/dl) was associated with an increased risk of CV mortality and all-cause mortality. Due to the nature of this study, the effect of underlying conditions like CKD and advanced age was not ruled out. Hypomagnesemia was not found to be associated with CV mortality.

Cheungpasitporn et al. (23) evaluated the association of dysmagnesemia and different outcomes in 65,974 hospitalized patients. Hypermagnesemia (>2.3 mg/dl) in comparison to hypomagnesemia (Mg <1.7 mg/dl) was a stronger predictor for poor outcomes including hospital mortality.

Several studies suggested a link between post-renal transplantation hypomagnesemia and new-onset diabetes (15, 16). Huang et al. conducted a retrospective study with 948 renal transplant recipients and observed 182 new-onset diabetes events. Multivariate analysis showed an increased risk between hypomagnesemia [<0.74 mmol/liter (1.8 mg/dl)] and new-onset diabetes (HR 1.24 per 0.1 mmol/liter decrease, 95% CI 1.05–1.46, *p* = 0.01) (15). Osorio et al. (24), performed a retrospective study with 589 renal transplant patients. Patients who received CNI had lower levels of Mg, and particularly, patients in the tacrolimus group had the lowest Mg level and the highest incidence of NODAT, but there was no relationship between Mg levels and occurrence of NODAT. Santos et al. (25) published a retrospective cohort study with 205 kidney recipients. The study did not find an association between hypomagnesemia and NODAT at a follow-up period of 1 year. Our study was not designed for evaluation of diabetes; however, no association between TWA of sMg at 6 months and glucose levels at that time point was observed.

Our study showed that hypomagnesemia post-transplantation was associated with a lower risk for all-cause mortality and MACE. Cheddani et al. (18) compared mortality risk in kidney transplant recipients beyond 1 year after successful transplantation vs. eGFR-matched CKD patients. In multivariable analysis, kidney transplant recipients had a 2.7-fold greater risk of mortality. Cardiovascular death rates in kidney transplant recipients (29.0%) approximated those of CKD patients (22.5%), whereas death rates due to infections were higher in kidney transplant recipients (19.4 vs. 10.0%). Our results demonstrate a higher mortality rate due to infections as mentioned above. It should be noted that renal transplant recipients within the lower quadrant of sMg at 6 months had better renal function compared to patients with higher levels of Mg. Although multivariate analysis including eGFR at 6 months did not change the association between low sMg and reduced risk of MACE, the difference in renal function might still explain the lower risk, as eGFR might poorly represent the full spectrum of kidney function. In addition, other unmeasured confounders might also play an important role in this association.

The difference between our results and other reports in different populations might be that after kidney transplantation sMg is mainly affected by immunosuppressive medications and kidney function (26), while in the general population, it is usually associated with a healthier diet. As a result, low sMg in the general population indicates poor nutrition, while in patients after kidney transplantation, it might indicate better kidney function and adequate immunosuppression.

In addition, our results strongly suggest that Mg supplementation was associated with higher all-cause mortality and MACE. As patients with low sMg, the main indication for Mg supplementation had a lower rate of adverse outcomes; these results do not support the routine use of Mg supplementation after kidney transplantation. It should be noted that very limited prospective interventional trials with patient-centered outcomes evaluating Mg supplementation are available. Such trials are urgently needed in order to make evidence-based decisions regarding the need for Mg supplementation.

This study has several important strengths. The use of all available sMg levels and time varying exposure produces a very precise evaluation of the exposure. In addition, the outcome analysis was based on detailed reports, each read by the researchers. Furthermore, the median follow-up time of more than 5 years enables adequate evaluation. In addition, we used several statistical methods to evaluate the risk of our primary and secondary outcomes and to increase the internal validity of our results.

Our study also has several limitations. Being a single center treating mostly a Caucasian population, the generalizability is limited. Also, despite extensive multivariate analysis, the possibility of residual confounding is still significant. Possible confounders might include kidney function and medications given over time as our analysis included creatinine and medications only at a fixed time point at 6 months post-transplant. Furthermore, eGFR is only rough estimation of true kidney function, and therefore, adjustment for kidney function might not be complete. Another possible confounder is nutrition status that might influence both sMg and CVD. In addition, the sample size, which is adequate for evaluation of the composite primary outcome, did not enable good evaluation of subgroups and the separate components of MACE. Also, the number of cardiovascular events in the study was small. In addition, evaluation of Mg supplementation was done only for one time point and dosing was not available. Detection bias is another possible limitation as high sMg was associated with reduced kidney function, which makes coronary angiography and the detection of CAD less likely.

## Conclusions

This study found an association between low exposure to sMg and better outcomes, which might reflect the confounding effect of better kidney function and adequate immunosuppression. Mg supplementation at 6 months post-transplantation was associated with an increased risk of all-cause mortality and MACE. This is the first study investigating the association between Mg levels and outcomes such as all-cause mortality and MACE in renal recipients. More studies, especially randomized controlled trials, are needed to further evaluate this important issue.

## Data Availability Statement

The raw data supporting the conclusions of this article will be made available by the authors, without undue reservation.

## Author Contributions

IL: participated in research design, performance of the research, data analysis and in the writing of the paper. TS: participated in performance of the research, interpretation of data for the work and in the writing of the paper. BR-Z: participated in research design, in the performance of the research, in data analysis and in in the revising of the paper. RR: participated in research design, in interpretation of data for the work and in the revising of the paper. MM: participated in research design, in the performance of the research and in the revising of the paper. All authors contributed to the article and approved the submitted version.

## Conflict of Interest

The authors declare that the research was conducted in the absence of any commercial or financial relationships that could be construed as a potential conflict of interest.

## Abbreviations

BMI: body mass index
CHF: congestive heart failure
CI: Confidence Interval
CKD: chronic kidney disease
CNI: calcineurin inhibitors
CV: cardiovascular
CVA: cerebrovascular accident
CVD: cardiovascular disease
eGFR: estimate glomerular filtrate rate
HBA1C: hemoglobin A1C
HDL: High-density lipoprotein
HR: Hazard Ratio
IHD: Ischemic Heart Disease
IQR: interquartile range
LDL: low-density lipoprotein
MACE: major cardiovascular outcome
Mg: magnesium
MI: myocardial infarction
PPI: proton-pump inhibitors
PVD: peripheral vascular disease
WBC: white blood cells
sMg: serum magnesium
TWA: time weighted average.
