# Supplementary material for: The Association Between Exposure to Low Magnesium Blood Levels After Renal Transplantation and Cardiovascular Morbidity and Mortality
Source: Front Med (Lausanne). 2021 Jul 12;8:690273. doi: 10.3389/fmed.2021.690273 (PMC8310919; doi:10.3389/fmed.2021.690273)
Supplement: Supplementary file 1 [file Data_Sheet_1.docx]

**The association between exposure to low magnesium blood levels after renal transplantation and cardiovascular morbidity and mortality**

ItayVahav M.D. ^c^ *, Tali Steinmetz M.D.^a.c^ *, Maya Molcho M.D. ^c^, Netta Lev M.D.^a,c^, TimnaAgur M.D.^a,c^, Eviatar Nesher M.D. ^b,c^, BenayaRozen-ZviM.D.^a,c^ and Ruth Rahamimov M.D. ^a,b,c^

1. Department of Nephrology, Rabin Medical Center, PetachTikva, Israel
2. Department of Organ Transplantation, Rabin Medical Center-Beilinson Hospital, PetachTikva, Israel
3. Sackler School of Medicine, Tel Aviv University, Tel Aviv, Israel

* IV and TS contributed equally to this work.

Supplementary material

Supplementary tables

Table S1 - The full model of the multivariate analysis that analyzed the association between sMg quartiles and the primary composite outcome of MACE and overall death.

| **Quartile cv+ mortality** | **Hazard Ratio** | **95% Confidence interval** | | **P value** |
| --- | --- | --- | --- | --- |
| Age (per year) | 1.059 | 1.036 | 1.082 | .000 |
| DM | 1.884 | 1.268 | 2.797 | .002 |
| History of IHD_ | 1.291 | .888 | 1.875 | .181 |
| BMI (per kg/m^2^) | 1.065 | 1.028 | 1.104 | .000 |
| Current Smoking | 2.033 | 1.307 | 3.162 | .002 |
| Mg supplementation | 2.064 | 1.214 | 3.512 | .007 |
| Male Gender | 1.579 | 1.035 | 2.409 | .034 |
| Length of Dialysis (per month) | 1.008 | 1.004 | 1.013 | .000 |
| Donor Age | 1.000 | .987 | 1.013 | .976 |
| Cyclosporine (vs. Tacrolimus) | .976 | .487 | 1.955 | .945 |
| mTOR (vs. Tacrolimus) | .754 | .323 | 1.762 | .515 |
| Albumin | .513 | .304 | .865 | .012 |
| eGFR | 1.002 | .990 | 1.014 | .730 |

Table S2- Association between sMg quartiles and the primary composite outcome of MACE and overall death by repeated measures GEE analysis.

GEE- generalized estimating equations.

|  | **Univariate analysis** | | | **Multivariate analysis** | | |
| --- | --- | --- | --- | --- | --- | --- |
|  | **Odd Ratio** | **95% Confidence interval** | **P value** | **Odd Ratio** | **95% Confidence interval** | **P value** |
| **Quartile 1** | Ref | Ref | Ref | Ref | Ref | Ref |
| **Quartile 2** | 1.31 | 0.77-2.21 | 0.31 | 1.64 | 0.96-2.8 | 0.071 |
| **Quartile 3** | 1.22 | 0.72-2.05 | 0.452 | 1.34 | 0.78-2.31 | 0.283 |
| **Quartile 4** | 1.69 | 1.03-2.77 | 0.037 | 1.8 | 1.04-3.1 | 0.034 |

Table S3 - The full model of the multivariate analysis that analyzed the association between sMg quartiles and death censored MACE.

| **Quartile cv** | **Hazard Ratio** | **95% Confidence interval** | | **P value** |
| --- | --- | --- | --- | --- |
| Age (per year) | 1.045 | 1.022 | 1.070 | <.001 |
| DM | 1.983 | 1.259 | 3.124 | .003 |
| History of IHD_ | 2.271 | 1.487 | 3.470 | <.001 |
| BMI (per kg/m^2^) | 1.083 | 1.038 | 1.129 | <.001 |
| Current Smoking | 2.183 | 1.304 | 3.655 | .003 |
| Mg supplementation | 2.232 | 1.295 | 3.846 | .004 |
| Donor Age | .994 | .979 | 1.009 | .442 |
| Cyclosporine (vs. Tacrolimus) | .897 | .346 | 2.324 | .822 |
| mTOR (vs. Tacrolimus) | 1.008 | .351 | 2.896 | .988 |
| Albumin | .383 | .208 | .707 | .002 |
| eGFR (per ml/min/1.73m^2^) | 1.009 | .995 | 1.022 | .207 |

Table S4 - Sensitivity analysis using the highest sMg level that analyzed the association between sMg quartiles and primary outcome.

|  | **Univariate analysis** | | | | **Multivariate analysis** | | | |
| --- | --- | --- | --- | --- | --- | --- | --- | --- |
|  | **HR** | **95% CI for HR** | | **P value** | **HR** | **95% CI for HR** | | **P value** |
| **Quartile 1** | ref | ref | ref | ref | ref | ref | ref | ref |
| **Quartile 2** | 2.127 | 1.255 | 3.608 | 0.005 | 1.875 | 1.099 | 3.198 | 0.021 |
| **Quartile 3** | 2.199 | 1.292 | 3.743 | 0.004 | 1.745 | 1.006 | 3.024 | 0.047 |
| **Quartile 4** | 2.270 | 1.331 | 3.872 | 0.003 | 1.882 | 1.088 | 3.255 | 0.024 |

Table S5 - Sensitivity analysis including patients with less than six available sMg values and who developed MACE events within first 180 days.

|  | **Univariate analysis** | | | | **Multivariate analysis** | | | |
| --- | --- | --- | --- | --- | --- | --- | --- | --- |
|  | **HR** | **95% CI for HR** | | **P value** | **HR** | **95% CI for HR** | | **P value** |
| **Quartile 1** | ref | ref | ref | ref | ref | ref | ref | ref |
| **Quartile 2** | 1.171 | 0.750 | 1.826 | 0.487 | 1.242 | 0.787 | 1.961 | 0.351 |
| **Quartile 3** | 1.017 | 0.640 | 1.615 | 0.943 | 1.102 | 0.682 | 1.781 | 0.692 |
| **Quartile 4** | 1.528 | 1.001 | 2.334 | 0.050 | 1.585 | 1.011 | 2.486 | 0.045 |

Table S6 - The full model of the multivariate analysis that analyzed the association between sMg quartiles and all-cause mortality.

| **Quartile cv** | **Hazard Ratio** | **95% Confidence interval** | | **P value** |
| --- | --- | --- | --- | --- |
| Age (per year) | 1.086 | 1.053 | 1.119 | <.001 |
| DM | 2.404 | 1.391 | 4.157 | .002 |
| History of IHD_ | 1.250 | .766 | 2.037 | .372 |
| BMI (per kg/m^2^) | 1.074 | 1.029 | 1.121 | .001 |
| Current Smoking | 2.114 | 1.193 | 3.748 | .010 |
| Mg supplementation | 1.312 | .608 | 2.836 | .482 |
| Albumin | 0.359 | .188 | .685 | .002 |

Table S7 - Association of sMg level with death censored MACE was analyzed by competing risk analysis.

|  | **Univariate analysis** | | | | **Multivariate analysis** | | | |
| --- | --- | --- | --- | --- | --- | --- | --- | --- |
|  | **HR** | **95% CI for HR** | | **P value** | **HR** | **95% CI for HR** | | **P value** |
| **Quartile 1** | ref | ref | ref | ref | ref | ref | ref | ref |
| **Quartile 2** | 1.560 | 0.990 | 2.470 | 0.110 | 1.490 | 0.940 | 2.370 | 0.150 |
| **Quartile 3** | 1.030 | 0.640 | 1.670 | 0.910 | 1.020 | 0.610 | 1.690 | 0.960 |
| **Quartile 4** | 1.470 | 0.930 | 2.330 | 0.170 | 1.880 | 1.140 | 3.110 | 0.038 |

Table S8- Association between sMg quartiles and death censored MACE by repeated measures GEE analysis.

GEE- generalized estimating equations.

|  | **Univariate analysis** | | | **Multivariate analysis** | | |
| --- | --- | --- | --- | --- | --- | --- |
|  | **Odd Ratio** | **95% Confidence interval** | **P value** | **Odd Ratio** | **95% Confidence interval** | **P value** |
| **Quartile 1** | Ref | Ref | Ref | Ref | Ref | Ref |
| **Quartile 2** | 1.24 | 0.68-2.25 | 0.479 | 1.56 | 0.84-2.92 | 0.159 |
| **Quartile 3** | 1.01 | 0.55-1.87 | 0.966 | 1.25 | 0.65-2.4 | 0.512 |
| **Quartile 4** | 1.69 | 0.97-2.94 | 0.062 | 2.15 | 1.15-4.02 | 0.017 |

Table S9- Association between sMg quartiles and all-cause mortality by repeated measures GEE analysis.

GEE- generalized estimating equations.

|  | **Univariate analysis** | | | **Multivariate analysis** | | |
| --- | --- | --- | --- | --- | --- | --- |
|  | **Odd Ratio** | **95% Confidence interval** | **P value** | **Odd**  **Ratio** | **95% Confidence interval** | **P value** |
| **Quartile 1** | Ref | Ref | Ref | Ref | Ref | Ref |
| **Quartile 2** | 1.38 | 0.64-2.98 | 0.418 | 1.55 | 0.74-3.24 | 0.241 |
| **Quartile 3** | 1.9 | 0.93-3.89 | 0.081 | 1.74 | 0.85-3.55 | 0.127 |
| **Quartile 4** | 2.18 | 1.08-4.41 | 0.029 | 1.94 | 0.94-4.02 | 0.074 |
